# Spectroscopic Study of Volatile Organic Compounds for the Assessment of Coffee Authenticity

**DOI:** 10.3390/molecules30173487

**Published:** 2025-08-25

**Authors:** Arianna Elefante, Marilena Giglio, Lavinia Mongelli, Adriana Bux, Andrea Zifarelli, Giansergio Menduni, Pietro Patimisco, Andrea Caratti, Cecilia Cagliero, Erica Liberto, Chiara Cordero, Luciano Navarini, Vincenzo Spagnolo, Angelo Sampaolo

**Affiliations:** 1 Consiglio Nazionale delle Ricerche (CNR), Istituto di Fotonica e Nanotecnologie, 70126 Bari, Italy; arianna.elefante@cnr.it; 2 PolySense Lab, Dipartimento Interateneo di Fisica, University and Politecnico of Bari, Via Amendola 173, 70126 Bari, Italy; l.mongelli4@phd.poliba.it (L.M.); a.bux21@studenti.uniba.it (A.B.); andrea.zifarelli@uniba.it (A.Z.); giansergio.menduni@poliba.it (G.M.); pietro.patimisco@uniba.it (P.P.); vincenzoluigi.spagnolo@poliba.it (V.S.); angelo.sampaolo@poliba.it (A.S.); 3 Dipartimento di Scienza e Tecnologia del Farmaco, Università di Torino, Via Pietro Giuria 9, 10125 Torino, Italy; andrea.caratti@unito.it (A.C.); cecilia.cagliero@unito.it (C.C.); erica.liberto@unito.it (E.L.); chiara.cordero@unito.it (C.C.); 4 Illycaffè S.p.A., Via Flavia 110, 34147 Trieste, Italy; luciano.navarini@illy.com

**Keywords:** coffee analysis, FTIR, pyridine, pyrazine, furan

## Abstract

This study aimed at defining the infrared spectral signatures of volatile organic compounds (VOCs) of relevant interest for coffee bean authentication and quality control. Fourier Transform Infrared Spectroscopy was employed to acquire the mid-infrared absorption spectra of some representative coffee markers, namely Pyridine, 2-Methylpyrazine, 2,5-Dimethylpyrazine, Furfural, 5-Methylfurfural and Furfuryl Alcohol, with high resolution of 0.1 cm^−1^. Mixtures of these VOCs simulating their amount in coffee seeds were analyzed using multilinear regression. The achieved results demonstrate the potentiality of coffee fingerprinting by VOC’s signature in the absorption spectra for discriminating coffee origin.

## 1. Introduction

Coffee authentication and quality control is addressing an increasing importance, being that coffee is among the main traded crops and one of the most consumed beverages worldwide. Many coffee studies rely on the analysis of its volatile chemical composition, which is intrinsically related to the sensed aroma. The coffee volatile organic compounds (VOCs) matrix is the result of several cascade processes determining the final sensorial profile. The chemical composition of green beans includes carbohydrates (59–61%), proteins (10–16%), lipids (11–17%), phenolic compounds (6–10%), minerals (4%), fatty acids (2%), caffeine (1–2%) and trigonelline (1%), among others [1]. However, several factors, including species and cultivars of coffee and other parameters related to geographical origin, such as climate, soil, temperature, and altitude, lead to distinguishable final products [2]. Starting from the seed non-volatile precursors, the chemical reactions occurring during processing, storage and, most importantly, roasting produce hundreds of identified volatile compounds belonging to different chemical classes including furans, pyrazines, pyridines, aldehydes, ketones, phenols, and sulfur compounds.

The state-of-the art techniques for the analysis of the complex and variable coffee VOCs matrices include Gas-Chromatography Mass-Spectrometry (GC–MS) and Fourier Transform Infrared Spectroscopy (FTIR) [3,4,5,6]. They offer application-oriented coffee fingerprinting based on the extraction of a set of characteristics that univocally identify, classify and discriminate coffee samples. As a preliminary study to the development of an optical sensor for coffee authenticity assessment, FTIR was employed to acquire high-resolution IR spectra of a set of compounds simplifying the complexity of coffee volatiles. Six molecules have been selected in this study as fingerprinting set, based on both their abundance and discriminating power in coffee authentication and quality control [2,7,8,9]. These molecules include Pyridine (P), 2-Methylpyrazine (2MP), 2,5-Dimethylpyrazine (2,5-DMP), Furfural (F), 5-Methylfurfural (5MF), and Furfuryl Alcohol (F-AL). Their typical abundance in the roasted coffee seeds is reported in Table 1 [10].

Pyridine is generated during roasting from the degradation of the trigonelline. 2-Methylpyrazine and 2,5-Dimethylpyrazine are the most abundant methylpyrazines in roasted coffee, generated from Maillard reactions and Strecker degradation of amino acids and reducing sugars. Furfural, 5-Methylfurfural and Furfuryl Alcohol are the higher content representatives of the furan derivatives, generated from the thermal degradation of sucrose and carbohydrates.

The six selected molecules (reported in Table 1) contribute to the aroma profile of the coffee, each of them alone eliciting a particular sensory note and some of them showing low odor threshold. Several studies employing GC–MS investigated the correlation of these VOCs quantity with the sensory analysis of the beans [11,12], revealing the possibility of monitoring the coffee roasting process to achieve the desired aroma profile [13]: positive correlation between roasting degree and levels of generated Pyrazine has been demonstrated, using the ratio between 2-Methylpyrazine and 2,5-Dimethylpyrazine at different time and temperature to distinguish weak roasting, leading to poor aromatic quality of the beans, and over-roasting, leading to the risk of having burned coffee beans [7]; the content of Pyridine was used to investigate the effect of different types of bean roasters [14].

Similar studies have been performed to assess the content of furan derivatives under different degrees of coffee roasting. In this case the special focus was not only on determining their contribution to the aroma profile, but mainly on controlling their levels, since they are associated with potential harmful effects [15,16]. According to the literature data, roasted coffee beans are the food that presents the highest amounts of furan derivatives compared to other solid food [17]. In particular, increasing attention is addressed to Furfuryl Alcohol since it can become a DNA-reactive substance with a mutagenic effect, leading the World Health Organization (WHO) International Agency for Research on Cancer (IARC) to set an acceptable daily intake of 0–0.5 mg/kg body weight [18]. Together with Furfural and 5-Methylfurfural, Furfuryl Alcohol has been found among the most abundant furans in coffee, encouraging studies on the variation in their content under different degrees of coffee roasting [8] to estimate the associated health risk and use them as rising time–temperature indicators [19].

Besides being markers of the roasting process, the six selected VOCs are reported in most of the studies on the discrimination of coffee species with different geographical origins. In particular, Toledo et al. [9] applied discriminant analysis to literature data from six published articles, identifying Pyridine and 2-Methylpyrazine as the most discriminating compound for coffee geographical origins, explaining 97.3% of the variance. 5-Methylfurfural, Furfuryl Alcohol and Furfural have also been reported as discriminant aroma markers related to geographical origin [2,20].

In this work, the acquisition of high-resolved absorption spectra of the selected VOCs and mixtures of VOCs at the vapor phase has been performed using the transmission FTIR. Coffee odorant mixtures simulating the volatile composition of coffee beans for the selected target analytes from different geographical origins have been analyzed to investigate the capability to discriminate the coffee origins by the analysis of the spectral features of the six selected VOCs. The scientific publication of Mondello et al. [21] was used as reference. They applied HS-SPME–GC–MS to the analysis of coffee blends with six different geographical origins: three roasted Arabica coffee beans from El Salvador, Costa Rica, and Santos and three roasted Robusta coffee beans from Togo, India, and Vietnam. Their results in the quantification of the six selected VOCs in each coffee blends were used in this work to realize reliable mixtures resembling the composition of the different geographical origins.

Successively, other mixtures simulating the variation in a single component have been prepared and acquired. Differently from the standard FTIR analysis aiming at samples’ classification or clustering, without a precise discrimination and quantification of single compound in the mixture, we applied multi-linear regression (MLR) in both cases to estimate the absolute content of each molecule starting from single-molecule reference.

The main objectives of this preliminary study consist in the: (i) consolidation of a protocol for generating reference spectra of individual molecules that ensures a high degree of reproducibility, (ii) acquisition of extended high-resolution IR spectra to enable, in an advanced research scenario, the identification of spectral regions suitable for discriminating a specific molecular pattern maximizing sensitivity and selectivity, (iii) detection and analysis, in the context of a multilinear regression, of any mismatches between expected and calculated concentrations of certain components, highlighting potential challenges in the generation of multi-component gas mixtures.

## 2. Materials and Methods

### 2.1. Materials

The following pure standard compounds were supplied by Sigma-Aldrich (Merck Life Science S.r.l., Milan, Italy): Pyridine (ACS reagent, ≥99.0%, CAS no. 110-86-1), 2-Methylpyrazine (≥99%, CAS no. 109-08-0), 2,5-Dimethylpyrazine (98%, CAS no. 123-32-0), Furfural (99%, CAS no. 98-01-1), 5-Methylfurfural (ReagentPlus^®^, 99%, CAS no. 620-02-0) and Furfuryl Alcohol (98%, CAS no. 98-00-0). A microsyringe (Hamilton, series 7000, Hamilton Company, Mumbai, India) with a capacity of 500 nL and precision of 5 nL was used to inject known volumes of liquid into the experimental setup for the acquisition of the absorption spectra. Mixtures were generated through subsequent injections of single standard liquid with the same syringe.

### 2.2. Experimental Setup

**Figure 1:**
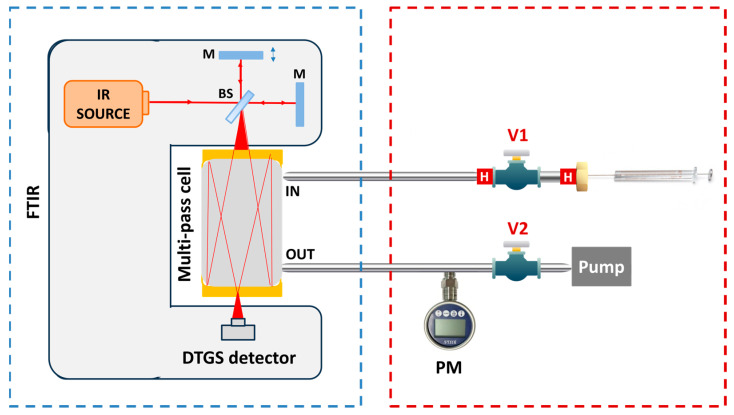
Experimental setup for the absorption spectra acquisition (blue box) and the sample preparation (red box). V1, V2: needle valve; PM: pressure meter; H: heater; BS: beam splitter; M: mirror.

The experimental setup employed for the sample preparation and the acquisition of the absorption spectra of the VOCs of interest is reported in Figure 1 and consists of two blocks, one for the sample preparation and one for the spectra acquisition.

#### 2.2.1. FTIR Spectrometer

Figure 1 reports the scheme of the instrumental setup. The first block in the blue box includes the Nicolet IS50 FTIR spectrometer (Thermo Fisher Scientific, Waltham, MA, USA) used to acquire the absorption spectra of the molecules. The FTIR employs the Polaris mid-IR broadband source coupled with a DTGS detector, enabling the investigation of the [400, 4000] cm^−1^ spectral range. The gas cell is a 200 mL multi-pass system with a 2 m optical path length. The cell inlet is connected through the valve V1 to the sample preparation block of the setup; the outlet is connected through the valve V2 to a pressure meter used to monitor the cell internal pressure and to the pump, used to evacuate the cell and clean it between different measurements.

The acquisition of the absorption spectra was carried out using the OMNIC Spectra v9.2 software by Thermo Fisher, setting the optimal spectral resolution of 0.1 cm^−1^ and averaging over 8 scans. The acquired spectra were analyzed using a MATLAB (R2024b)-based software.

#### 2.2.2. Sample Preparation

The VOCs of interest are supplied in the liquid phase at atmospheric temperature and pressure, requiring a custom sample evaporation method to obtain vapor-phase absorption spectra. Table 2 reports the normal boiling points, falling in the [113, 170] °C range, and the normal vapor pressure, falling in the [0.4,19]. Torr range, for the VOCs of interest.

The procedure adopted to ensure the repeatable vaporization of the standard liquid samples is based on the Clausius–Clapeyron equation [23], which describes the exponential increase in the molecule vapor pressure with temperature during the phase transition of a single constituent. The predicted trends for the target molecules indicate that the complete sample evaporation can be achieved by increasing the system temperature to ~100 °C to raise vapor pressure, and reducing the gas pressure below ~100 Torr to lower the boiling point.

The vapor pressure reported in Table 2 demonstrates that the proposed strategy provides feasible temperature and pressure conditions to achieve efficient sample preparation while avoiding unwanted thermal degradation. The section of the setup used to implement this procedure is outlined with a dashed red box in Figure 1. It consists of a 2 cm long stainless-steel tube connected on one side to the inlet of the cell through the valve V1 and closed with a septum nut on the other side. The tube is heated using micro heaters up to ~100 °C. The valve V1 is initially opened and the FTIR pump is used to reach a vacuum level pressure within the tube. Then, V1 is closed, and a known volume of standard liquid is injected into the tube through the septum nut using a µL syringe. The combined effect of lowering the pressure and increasing the temperature promotes the total evaporation of the liquid inside the tube. V1 is then opened, enabling the gas produced by evaporation to flow into the cell. To prevent the recondensation of the vapor phase, the delivery line is made of stainless-steel tubing and heated with resistors, while the cell is maintained at 120 °C.

### 2.3. Experimental Procedure

An analysis of individual VOCs were first performed, starting with a test of the repeatability of both the evaporation and measurement processes. A known volume of each VOC in liquid phase was totally evaporated, and the corresponding spectrum was acquired. After the acquisition, the pump of the FTIR system was used to purge the cell, and the process was repeated for the same molecule at the same volume to assess the evaporation and spectral reproducibility. Then, calibration curves were obtained for each VOC by acquiring FTIR spectra at progressively increasing injected volumes listed in Table 3.

Mixtures were generated by injecting the six VOCs into the cell, one at a time. The corresponding absorption spectrum was recorded every 2 min, allowing stabilization between two successive injections. The injected volumes used to simulate the geographical origin of the seeds are listed in Table 4.

For the second analysis, focused on varying a single component within a fixed matrix, the 5-component fixed matrix was prepared by sequentially injecting VOCs into the cell. The injected volumes corresponded to the mean value (MV) of the VOC content across the six geographical mixtures listed in Table 4. The sixth variable VOC was then added in three concentrations: (i) 50% of the MV, (ii) equal to the MV, and (iii) 50% above the MV. Absorption spectra were acquired at each concentration level. This procedure was repeated alternating the variable compound among Pyridine, 2-Methylpyrazine and Furfuryl Alcohol.

## 3. Results and Discussion

### 3.1. Repeatability of the Evaporation Procedure and Measurements

**Figure 2:**
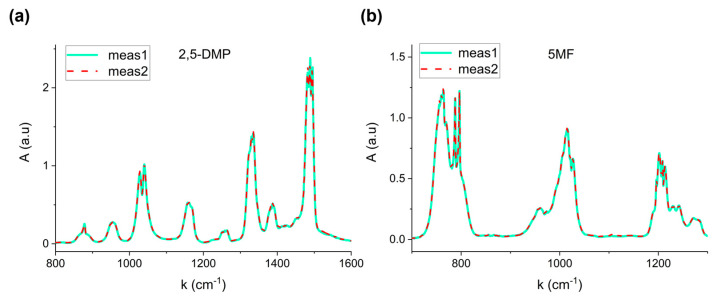
Repeatability of measurements at fixed volume for (**a**) 2,5-Dimethylpyrazine (1 µL) and (**b**) 5-Methylfurfural (2 µL).

Figure 2 shows two representative absorption spectra corresponding to two distinct evaporation processes of 1 µL of 2,5-Dimethylpyrazine (a) and 2 µL of 5-Methylfurfural (b).

The perfect overlap confirms the reproducibility of the procedure, proving that the total evaporation system based on the heated steel tube significantly avoids recondensation effects, thereby allowing calibration reliability. The relative standard error on three repeated measurements resulted lower than 4% for the six VOCs.

### 3.2. VOCs Reference Spectra and Calibration

**Figure 3:**
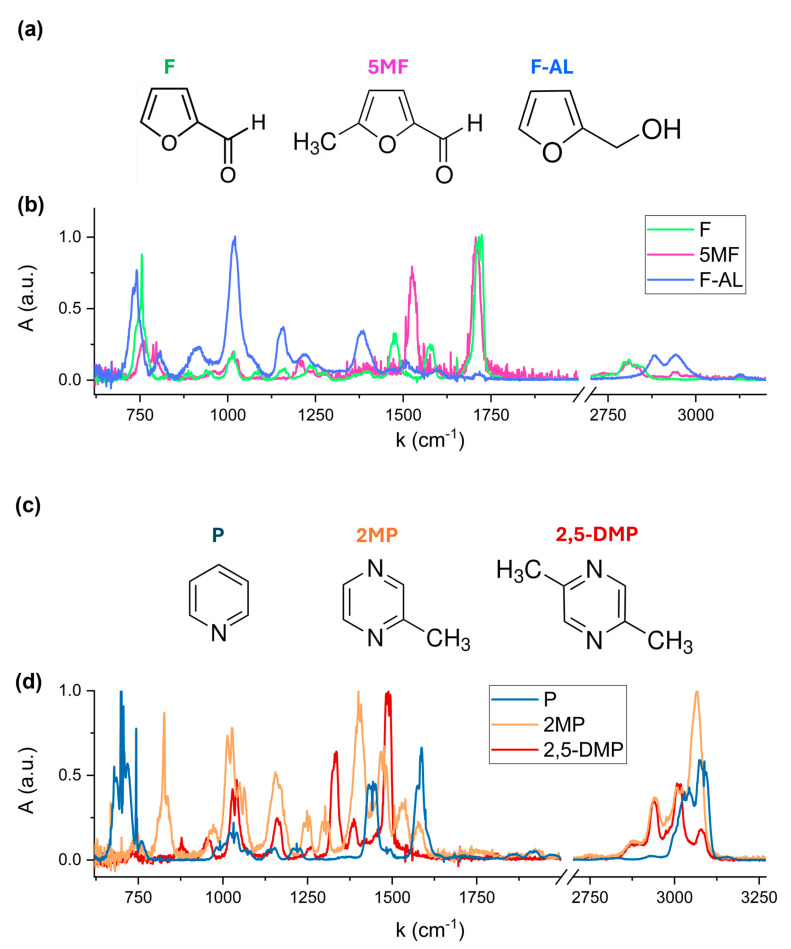
Molecular structures of Furfural, 5-Methylfurfural and Furfuryl Alcohol (**a**) and Pyridine, 2-Methylpyrazine and 2,5-Dimethylpyrazine (**c**), and the corresponding FTIR absorption spectra (**b**) and (**d**).

Reference spectra of individual VOCs were acquired to accurately identify the characteristic spectral features of compound. Figure 3b,d show the absorption spectra of the six VOCs, each normalized to its maximum absorbance peak. The spectral region [2000, 2700] cm^−1^ has been excluded, as none of the analyzed compounds exhibits significant absorption in this region. Distinct spectral signatures are clearly observed, corresponding to the unique molecular structures of each VOC. Furfural, 5-Methylfurfural, and Furfuryl Alcohol are classified as furanoids, which are heterocyclic compounds containing a furan ring, a five-membered aromatic structure containing oxygen atom (see Figure 3a). These molecules differ in their functional groups: Furfural contains an aldehyde (-CHO) group; 5-Methylfurfural adds a methyl (-CH_3_) group; and in Furfuryl Alcohol, the aldehyde is replaced by a hydroxymethyl (-CH_2_OH) group. Pyridine and pyrazine-derivatives are aromatic heterocyclic compounds with a six-membered ring containing one and two nitrogen atoms, respectively, as shown in Figure 3c. The addition of one and two methyl (-CH_3_) substituent groups to the Pyrazine ring results in the formation of 2-Methylpyrazine and 2,5-Dimethylpyrazine structures, respectively.

The absorption bands observed in the 720–770 cm^−1^ region in Figure 3b are associated with the out-of-plane C-H bending vibrations within the furan ring of Furfural, 5-Methylfurfural and Furfuryl Alcohol. The exact spectral position and relative intensity of these bands depends on the substituent attached to the ring. The most intense peak of Furfuryl Alcohol at 1010–1022 cm^−1^ is assigned to the C-O stretching vibration of the hydroxymethyl (-CH_2_OH) functional group. The distinct absorption band in the 1700–1720 cm^−1^ region corresponds to the carbonyl C=O stretching of the aldehyde (-CHO) functional group present in Furfural and 5-Methylfurfural. A shift in this band is due to the presence of the methyl (-CH_3_) substituent in Furfural, which modifies the electronic environment of the carbonyl bond.

Pyridine exhibits its most intense fingerprint feature between 694 and 704 cm^−1^, corresponding to out-of-plane C-H bending vibrations of the aromatic ring. 2-Methylpyrazine and 2,5-Dimethylpyrazine exhibit characteristic absorption features at 1010–1034 cm^−1^, attributed to C-N and C-C vibrations within the pyrazine ring, with a slight shift in band position due to the presence of two methyl groups in 2,5-Dimethylpyrazine.

Calibration spectra at different injected volumes exhibit a linear increase in the signal intensity with volume for each molecule, as shown in the Supplementary Materials—Section S1, panel (a) of Figures S1–S6. For each VOC, the calibration curve was obtained by selecting a distinct absorption band and plotting the integrated peak area as a function of the molecule injected volume. A linear fit was then performed, as shown in the Supplementary Materials—Section S1, panel (b) of Figures S1–S6. Table 3 summarizes the spectral integration range, the calibration volumes and the fit results for each molecule, showing R-squared values higher than 0.991.

**Table 3:** Spectral integration region, volume range and linear fit results for each molecule.

Molecule	Volume Range (nL)	Integration Range (cm^−1^)	Slope (a.u./nL)	Intercept (a.u.)	R^2^
P	200–1000	2889–3194	0.0351 ±0.0006	1.54 ± 0.41	0.999
2MP	100–200	2800–3170	0.0335 ± 0.0003	0.07 ± 0.04	0.999
2,5-DMP	25–100	2815–3140	0.063 ± 0.002	0.550 ± 0.103	0.998
F	30–150	1650–1800	0.125 ± 0.002	0.745 ± 0.202	0.999
5MF	10–50	1666–1755	0.098 ± 0.007	−0.08 ± 0.21	0.991
F-AL	30–120	850–1100	0.073 ± 0.002	0.22 ± 0.16	0.998

### 3.3. FTIR Analysis of Coffee Odorant Mixtures Simulating Different Geographical Origin

Further analyses have been conducted to evaluate the capability to distinguish and quantify VOCs within complex coffee–odorant matrices representing different geographical origins of the coffee bean. Six simulated mixtures, corresponding to six distinct origins, were prepared using the nominal volumes reported in Table 4. Details on the calculation of the volumes using literature data are reported in the Supplementary Material—Section S2, Tables S1–S4.

**Figure 4:**
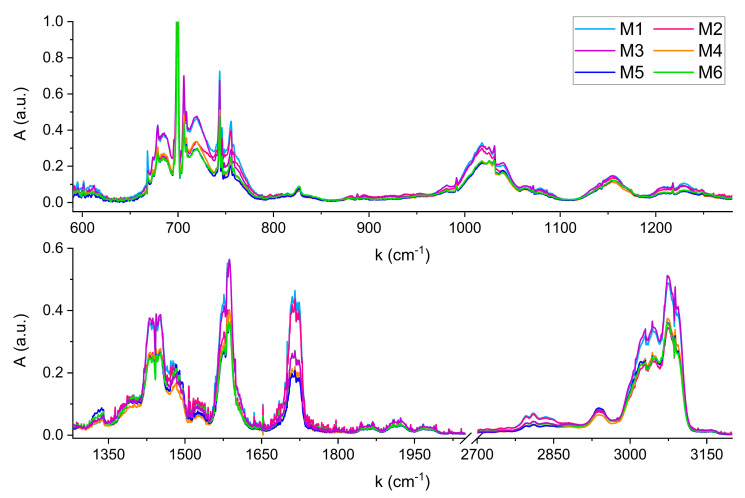
FTIR spectra of the six mixtures simulating coffee samples with different geographical origins.

Figure 4 shows the FTIR spectra acquired for the six mixtures.

**Table 4:** Nominal (N) and calculated (MLR) volumes of each molecule in the six mixtures.

	M1		M2		M3	
Molecule	N vol. (nL)	MLR vol. (nL)	N vol. (nL)	MLR vol.	N vol. (nL)	MLR vol. (nL)
P	944 ± 7	927.6 ± 11.7	635 ± 7	639 ± 9	971 ± 7	951.7 ± 0.2
2MP	139 ± 5	140.0 ± 0.3	124 ± 5	122 ± 0.5	121 ± 5	120.7 ± 0.2
2,5-DMP	47 ± 5	41.4 ± 0.6	57 ± 5	56 ± 0.3	52 ± 5	55.5 ± 0.8
F	120 ± 5	108.9 ± 0.2	107 ± 5	99 ± 0.1	60 ± 5	57.3 ± 0.3
5MF	31 ± 5	25.2 ± 0.3	30 ± 5	29 ± 0.03	20 ± 5	17.5 ± 0.3
F-AL	90 ± 5	71.4 ± 0.2	100 ± 5	83 ± 0.2	106 ± 5	82.6 ± 0.6
	**M4**		**M5**		**M6**	
**Molecule**	**N vol. (nL)**	**MLR vol. (nL)**	**N vol. (nL)**	**MLR vol. (nL)**	**N vol. (nL)**	**MLR vol. (nL)**
P	680 ± 7	651 ± 0.2	620 ± 7	619 ± 0.2	570 ± 5	568.5 ± 0.2
2MP	125 ± 5	131 ± 0.7	167 ± 5	161.7 ± 0.3	177 ± 5	183 ± 0.3
2,5-DMP	50 ± 5	50.2 ± 0.2	96 ± 5	110 ± 0.2	87 ± 5	79 ± 0.2
F	44 ± 5	48 ± 0.1	44 ± 5	44.5 ± 0.1	60 ± 5	57.2 ± 0.1
5MF	10 ± 5	9.6 ± 0.1	13 ± 5	12.1 ± 0.1	14 ± 5	20.5 ± 0.1
F-AL	62 ± 5	51.9 ± 0.2	53 ± 5	37.7 ± 0.1	46 ± 5	34.6 ± 0.2

Compared to the reference spectra of single compounds, no shifts in wavenumbers due to anelastic collisions between the gas phase molecules in the mixtures were observed. Any shift, if present, is lower than the spectral resolution of 0.1 cm^−1^. From a qualitative perspective, the six FTIR spectra can be distinguished by comparing their characteristic absorption features across the wide infrared region. For example, a clear visual distinction can be made between mixtures with significantly different absorbance levels, such as M3 and M6. The higher absorbance of M3 in the regions around 1450 cm^−1^, 1580 cm^−1^, and 3000 cm^−1^, reflects the different content of Pyridine in M3 and M6, corresponding to 970 nL and 750 nL, respectively. Conversely, both mixtures exhibit overlapping features around 1720 cm^−1^, corresponding to the C=O stretching absorption of Furfural and 5-Methylfurfural, whose concentrations are nearly identical in the two samples (see Table 3). Instead, the spectral feature centered at 1720 cm^−1^ becomes useful for distinguishing mixtures with similar Pyridine content but differing levels of Furfural and 5-Methylfurfural, such as M2 and M4. Although these mixtures contain comparable Pyridine volumes (635 nL and 680 nL), resulting in similar absorbance in most of the infrared spectral range, their significantly different Furfural volumes (107 nL and 44 nL) result in a marked difference in absorbance at 1720 cm^−1^.

Beyond qualitative comparison, a quantitative estimation of the amount of the six VOCs in each mixture can be obtained by modeling each spectrum in Figure 4 as a linear combination of the six reference spectra shown in Figure 3. The linearity of the calibration curves shown in Table 3 allowed one reference spectra for each molecule to be used. The concentration of each molecule in the reference corresponds to the mean value of its variation range in the six mixtures: 700 nL for the Pyridine, 140 nL for the 2-Methylpyrazine, 65 nL for the 2,5-Dimethylpyrazine, 70 nL for the Furfural, 20 nL for the 5-MethylFurfural and 75 nL for the Furfuryl Alcohol.

A MATLAB R2024b software based on a multi-linear regression (MLR) algorithm was developed to retrieve the VOCs concentrations in all the six mixtures. The whole spectral range shown in Figure 4 was included in the analysis for each compound, since the six molecules present characteristic absorption features covering all the infrared region. The [2000, 2700] cm^−1^ range, not shown in Figure 3 and Figure 4, was excluded since the only significant absorption is due to CO_2_, which could introduce noise in the MLR estimations. Table 4 reports the nominal and calculated volumes of each molecule in the six mixtures, together with the corresponding standard errors. The error in nominal values accounts for a ±5 nL uncertainty due to syringe injection. For Pyridine, which was present at concentrations higher than 500 nL, two consecutive injections were performed, leading to a combined injection error of ±7 nL. The MLR errors represent the standard errors associated with the algorithm’s volume estimations.

The results reported in Table 4 have been used to evaluate the percentage discrepancy between the calculated and nominal concentrations (Δ), and the percentage standard errors (PSE) for each molecule in each mixture. The PSE is calculated as the ratio between the standard error, resulting from the propagation of the syringe injection and MLR estimation errors, and the nominal value (Table 5).

The most significant experimental evidence to focus on for the analysis and interpretation of the results is the discrepancy between the calculated and the nominal concentration for each mixture varies with the VOCs. The best agreement was found for the most abundant Pyridine and 2-Methylpyrazine, exhibiting a mean discrepancy lower than 2.5%, followed by Furfural and the 2,5-Dimethylpyrazine with mean values of 6% and 7.4%, respectively. 5-Methylfurfural shows the wider range of discrepancy among the six mixtures, ranging from 3% to 46%. Nevertheless, its absolute concentration in the six mixtures varies from ~10 nL to ~30 nL, with an injection error of ±5 nL due to the syringe precision. Consequently, the PSE ranges between 17% and 52%, which is comparable with the Δ variation range. Furfuryl Alcohol showed the worst agreement between nominal and calculated volumes, with a mean discrepancy of 21.8%, and with the calculated values lower than the nominal ones for all the mixtures. To explain these mismatches, the hypothesis to be verified through further research is that the missing volume is due to physical/chemical interactions with other components in the mixture.

**Figure 5:**
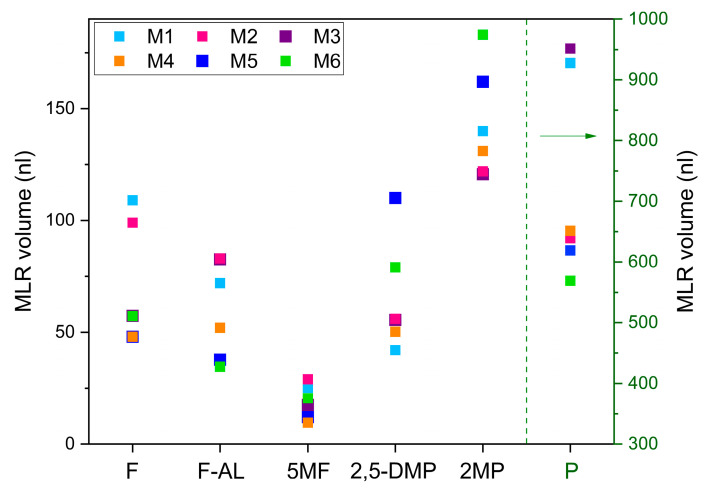
Trends of variation in each VOCs in the six mixtures, based on the volumes retrieved by the FTIR spectra analysis. The green dotted line and arrow indicate the volume scale used for Pyridine, shown on the right of the graph.

The retrieved VOCs estimation was investigated as a potential set of characteristics capable of uniquely identifying the different types of coffee. To this aim, Figure 5 illustrates the retrieved composition of each molecule in each mixture.

These quantitative results support the qualitative analysis discussed in relation to Figure 4, showing the capability to distinguish the mixtures based on the distinct variation patterns of the six VOCs. This means that the analysis of the FTIR spectra, although remains too broad and insufficiently specific at this stage to optimally discriminate individual molecules or small subsets of them, returns distinguishable patterns of VOCs’ concentration which could be used to discriminate coffee samples according to their geographical origin. Although returning distinguishable patterns, the MLR approach suffers from limitations when dealing with spectroscopic analysis of overlapping absorption structures of multi-component mixtures, which can result in lack of precision and accuracy due to the multicollinearity or the variables. A potential solution is the application of more sophisticated algorithms, like Partial Least Squares Regression [24,25].

### 3.4. Mixtures: Single Component Variation in a Fixed Matrix

A second analysis focused on VOC mixtures composed by a fixed matrix of five molecules at constant concentrations, while systematically varying the concentration of one target compound. Monitoring changes in individual VOCs is particularly relevant for quality control during the coffee roasting process [7,14]. The selected variable compounds were Pyridine, 2-Methylpyrazine and Furfuryl Alcohol.

**Figure 6:**
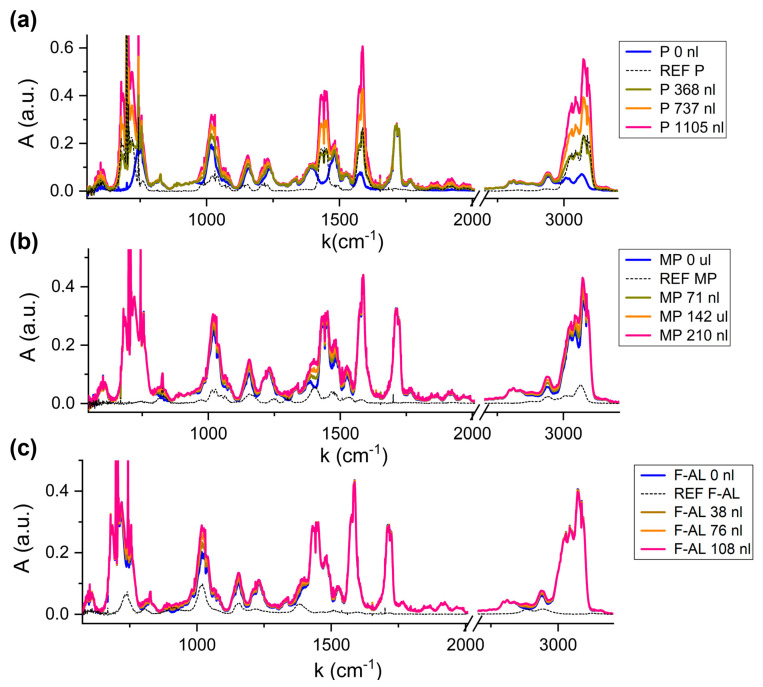
Spectra of mixture with increasing volumes of Pyridine (**a**), 2-Methylpyrazine (**b**) and Furfuryl Alcohol (**c**).

Figure 6 shows the absorption spectrum of the fixed matrix (blue line) and the spectra corresponding to increasing content of the variable compound (green, orange and pink lines), compared to the reference spectrum of the variable molecule (black dotted line), for Pyridine (a), Methylpyrazine (b) and Furfuryl Alcohol (c).

It is evident how the absorption features of Pyridine in Figure 6a are clearly affecting the overall spectrum profile, even when adding the lower volume (green line). The only structure which remains unchanged is the 1720 cm^−1^, associated with the absent carbonyl stretching.

Compared to the Pyridine variation, the 2-Methylpyrazine and Furfuryl Alcohol contribution to the matrix spectrum is less visible, due to two reasons. Firstly, the presence of Pyridine at the ~700 nL level in the fixed matrix affects all the spectrum. Secondly, the injection volumes of 2-Methylpyrazine (MV ~140 nL) and Furfuryl Alcohol (MV ~75 nL) are lower compared to the Pyridine ones. Nevertheless, the detection of these variable compounds is still possible targeting their fingerprinting regions. For 2-Methylpyrazine, in the regions around 825 cm^−1^, [1000, 1200] cm^−1^, 1440 cm^−1^ and 3000 cm^−1^ the characteristic shape of the 2-Methylpyrazine peaks influences the mixture, resulting in a new spectral configuration with increasing amplitude as the injected volume of the VOC increases. For Furfuryl Alcohol, the most intense absorption feature at 1010 cm^−1^, corresponding to the C-O stretching vibration of the hydroxymethyl functional group, as well as the structures at 1150 cm^−1^ or 1400 cm^−1^, slightly affect the fixed-matrix spectrum.

The MLR analysis was applied to the acquired spectra, returning the results summarized in Table 6.

As for the geographical discrimination application, good results are achieved for Pyridine and 2-Methylpyrazine, while the discrepancy for Furfuryl Alcohol detection is higher, with the estimation becoming worse with the increase in its concentration. This supports the hypothesis that furfuryl alcohol is the most critical molecule in terms of intermolecular interaction when introduced into a multi-component mixture.

## 4. Conclusions

In the context of coffee VOCs emission studies, high-resolution fingerprinting of roasted coffee is needed for the quality control authentication and traceability of premium quality products. From this perspective, this work investigates the importance of optical characterization of VOCs, particularly in the mid-IR range, for improving the quality and origin recognition based on the VOCs produced during roasting processes.

A reproducible and effective method to achieve the complete evaporation of Pyridine, 2-Methylpyrazine, 2,5-Dimethylpyrazine, Furfural, 5-Methylfurfural and Furfuryl Alcohol, selected as representative roasted coffee markers, was developed, allowing the acquisition of high-resolution IR spectra and the precise identification of their characteristic absorption bands. Multilinear regression was exploited to analyze coffee–odorant mixtures to evaluate its potential ability in discriminating the geographical origin of the coffee seeds based on the spectral signatures of their volatile composition and to appreciate variations in a single VOC in a fixed matrix. The geographical classification reached an overall accuracy of 90.7% considering all the six molecules, which improves to 95.6%, excluding the low abundant 5-Methylfurfural and Furfuryl Alcohol, confirming the potential exploitability of these VOCs’ spectra for applications in food authenticity and traceability. Further improvements to the classification accuracy can be investigated, in particular for the Furfuryl Alcohol estimation. An interesting possibility is implementing Partial Least Squares Regression to the analysis of the mixtures, training the algorithm with coffee odorant mixtures with different contents of Furfuryl Alcohol. This would help in modeling the system, obtaining a deeper understanding of the potential interaction among the mixture’s components.

The precise knowledge of the VOCs infrared spectral properties represents important basic instruments for future developments. Firstly, the acquisition of single-gas reference spectra allows the precise characterization of VOCs in coffee–odorant mixtures, enhancing progress in the comprehension of flavor development. Secondly, it provides the basic tool to properly design optical sensors by (i) identifying the spectral ranges of interest to maximize the accuracy and discrimination precision for a given subset of target molecules, (ii) selecting a laser source with a suitable tuning range, and (iii) determining the most effective spectroscopic approach for detection in terms of modulation techniques. These optical sensing platforms will be designed and developed aiming at real-time and in situ analysis, according to the operating conditions of the specific application scenario. The discrimination of VOCs through their spectral fingerprint can thus offer a valid alternative to the gold standard GC–MS, overcoming some limitations for real-time and at/in-line applications. As a further improvement for the analysis of complex mixtures, a GC column could be coupled upstream the optical platform to exploit a time/frequency double-domain discrimination system, where gas chromatography provides temporal separation of different compounds potentially overlapping in terms of IR-bands, while the optical sensor discriminates co-eluted substances thanks to the characteristics spectral signatures.

## Figures and Tables

**Table 1:** Mean concentration of Pyridine, 2-Methylpyrazine, Furfuryl Alcohol, Furfural, 5-Methylfurfural and 2,5-Dimethylpyrazine in the coffee bean and corresponding odor description and threshold (OT) [10].

Molecule	Mean µg/1 g of Coffee	Odor Description	OT (ppb)
P	50	Fishy, amine, bitter, roasted	77
2MP	25	Nutty	60,000
2,5-DMP	30	Nutty-roasted, cocoa, grassy	80
F	60	Sweet, woody, almond	280
5-MF	50	Spice, caramel, maple	6000
F-AL	300	Mild, slightly caramel-like	2

**Table 2:** Boiling points (T_boil_) and Vapor Pressure (VP) values for the VOCs of interest [22]. The vapor pressure of 5MF at 100 Torr was not estimated due to the missing value of the enthalpy of vaporization in the NIST database.

Molecule	T_boil_ (°C) at 760 Torr	VP (Torr) at 25 °C	VP (Torr) at 100 °C
P	113	19.0	490
2MP	146	7.2	246
2,5-DMP	168	3.2	106
F	143	2.3	102
5MF	165	0.7	/
F-AL	170	0.4	75

**Table 5:** Percentage discrepancy between nominal and calculated volume values (Δ) and relative standard error (PSE) for each VOC in each mixture. Average values ($\bar{\Delta }$ and $\bar{PSE}$) are also reported.

	M1		M2		M3		M4		M5		M6			
Molecule	Δ%	PSE%	Δ%	PSE%	Δ%	PSE%	Δ%	PSE%	Δ%	PSE%	Δ%	PSE%	$\bar{\Delta }\%$	$\bar{PSE}\%$
P	1.7	1.5	0.6	1.1	2.0	0.7	4.3	1.1	0.2	1.1	0.6	1.2	**1.6**	**1.1**
2MP	0.7	3.6	1.6	4.1	0.2	4.1	4.8	3.9	3.2	3.1	3.6	2.7	**2.4**	**3.6**
2,5-DMP	11.9	12.2	1.8	8.9	6.7	9.1	0.4	10	14.6	4.5	9.2	6.3	**7.4**	**8.5**
F	9.3	4.6	7.5	5.1	4.5	8.7	9.1	10.4	1.1	11.2	4.7	8.7	**6.0**	**8.1**
5MF	18.7	19.9	3.3	17.2	12.5	28.6	4	52.1	6.9	41.3	46.4	24.4	**15.3**	**30.6**
F-AL	21.5	7.0	17	6	22.1	6.1	16.3	9.6	28.9	13.3	24.8	14.5	**21.8**	**9.5**

**Table 6:** Percentage discrepancy between nominal and calculated volume values (Δ).

Target Compound	Δ (MV − 50%)	Δ (MV)	Δ (MV + 50%)
P	3.5%	7.7%	9.8%
2MP	0.7%	1.8%	0.2%
F-AL	5%	20.1%	22.2%

## Data Availability

Data are contained within the article and Supplementary Materials.

## Supplementary Materials

The following supporting information can be downloaded at: https://www.mdpi.com/article/10.3390/molecules30173487/s1, Figures S1–S6: Calibration of single VOCs; Tables S1–S4: Definition of volumes for the coffee-like matrix.

## Abbreviations

The following abbreviations are used in this manuscript:

2MP	2-Methylpyrazine
2,5-DMP	2,5-Dimethylpyrazine
5MF	5-Methylfurfural
F	Furfural
F-AL	Furfuryl Alcohol
FTIR	Fourier Transform Infrared
GC–MS	Gas-chromatography mass-spectroscopy
HS-SPME	Headspace solid-phase microextraction
P	Pyridine
PLSR	Partial Least Squares Regression
VOC	Volatile organic compound
