# Supplementary material for: Spectroscopic Study of Volatile Organic Compounds for the Assessment of Coffee Authenticity
Source: Molecules. 2025 Aug 25;30(17):3487. doi: 10.3390/molecules30173487 (PMC12430174; doi:10.3390/molecules30173487)
Supplement: Supplementary file 1 [file molecules-30-03487-s001.zip › molecules-3754327-supplementary.pdf]

molecules

MDPI

Supplementary Materials

S1. Calibration of single VOCs

The calibration of each molecule of interest was performed by acquiring FTIR absorption spectra at different liquid injection volumes. The following figures show the results for Furfural (Figure S1), 5-Methylfurfural (Figure S2), Furfuryl Alcohol (Figure S3), Pyridine (Figure S4), 2-Methylpyrazine (Figure S5), and 2,5-Dimethylpyrazine (Figure S6).

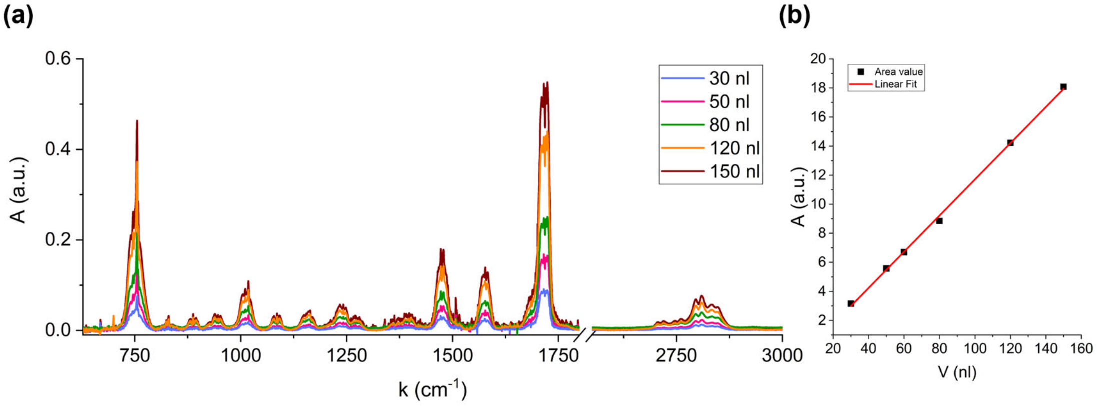

Figure S1. (a) FT–IR absorption spectra of Furfural acquired at different volumes; (b) Calibration curve of Furfural.

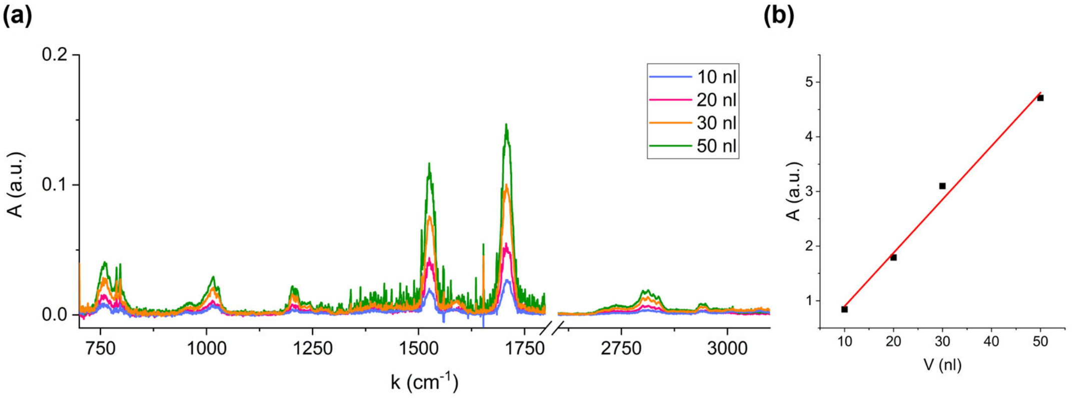

Figure S2. (a) FT-IR absorption spectra of 5-Methylfurfural acquired at different volumes; (b) Calibration curve of 5-Methylfurfural.

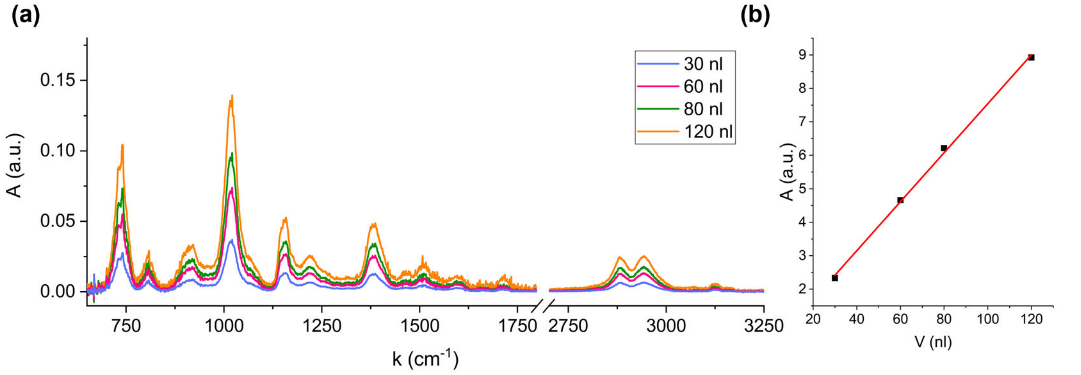

Figure S3. (a) FT-IR absorption spectra of Furfuryl Alcohol acquired at different volumes; (b) Calibration curve of Furfuryl Alcohol.

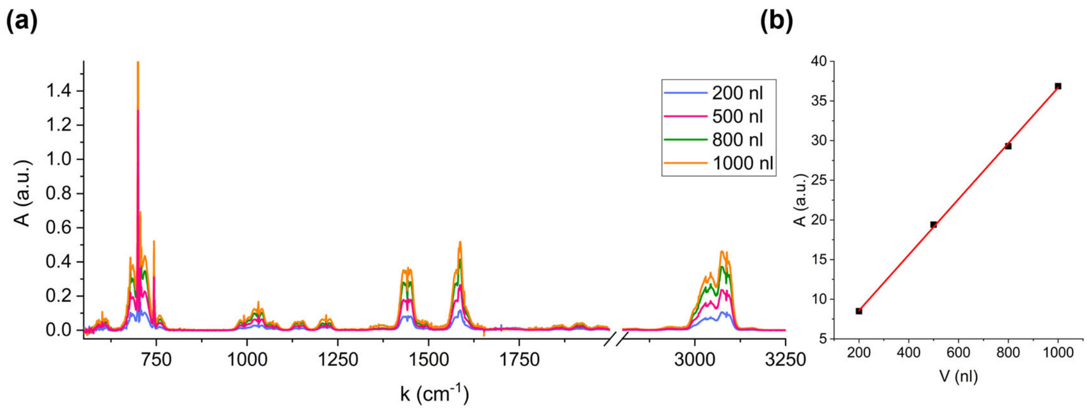

Figure S4. (a) FT-IR absorption spectra of Pyridine at different volumes; (b) Calibration curve of Pyridine.

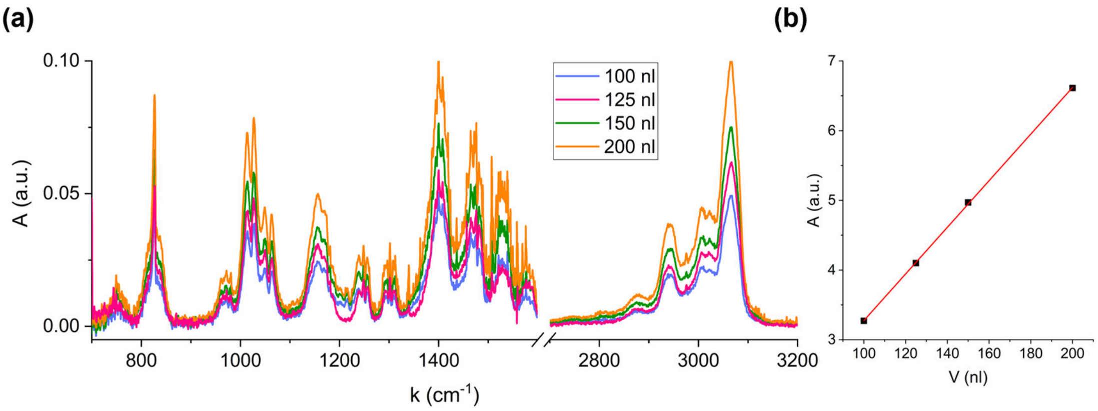

Figure S5. (a) FT-IR absorption spectra of 2-Methylpyrazine at different volumes; (b) Calibration curve of Methylpyrazine.

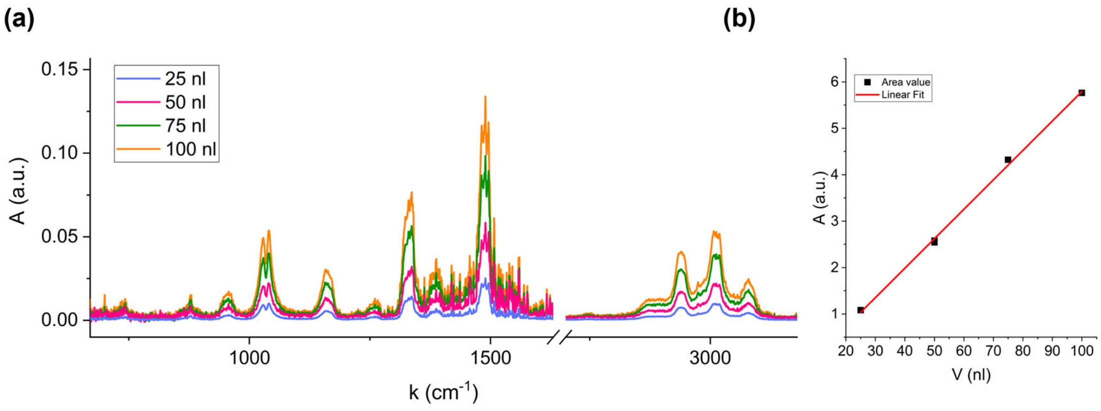

Figure S6. (a) FT-IR absorption spectra of 2,5-Dimethyl-pyrazine at different volumes; (b) Calibration curve of 2,5-Dimethylpyrazine.

S2. Definition of volumes for the coffee-like matrix

The estimation of the injection volumes to realize the coffee-odorant mixtures was based on the combination of: (i) the average composition of the selected VOCs in the coffee headspace; (ii) the VOCs content in real coffee blends with different geographical origins.

The average composition of the VOCs expected in the coffee headspace was calculated as follows. Table S1 reports in the 2nd column the mean concentrations of the VOCs in the solid coffee beans, expressed in μg/g of dry coffee, as reported by Flament [11]. A certain volatile fraction of every compound will be distributed in the coffee headspace; the volatile concentration can be estimated by multiplying the bean content by the ambient vapor pressure for each VOC (see Table 2), giving the results in the 3rd column of the table. Finally, the mean volumes are obtained by dividing the VOCs masses by the density of each molecule at 25 °C [22], as reported in the last column.

Table S1. Mean concentration of Pyridine, 2-Methylpyrazine, Furfuryl Alcohol, Furfural, 5-Methylfurfural and 2,5-Dimethylpyrazine in the coffee bean and in the coffee headspace.

Molecule	Mean μg/1g of coffee	Concentration in headspace (μg)	Mean Volume (MV) in headspace (nl)
Pyridine	50	950	**971**
2-Methylpyrazine	25	182	**177**
Furfuryl Alcohol	300	120	**106**
Furfural	60	139	**120**
5-Methylfurfural	50	34	**31**
2,5-Dimethylpyrazine	30	95	**96**

The expected volumes for each VOC in the different geographical blends were estimated using the data published by Mondello et al. (2004) as reference [21]. In their study, Mondello et al. applied headspace-solid-phase microextraction–gas chromatography–mass spectrometry (HS-SPME–GC–MS) to the analysis of six coffee blends with different geographical origins—three roasted Arabica coffee beans from El Salvador, Costa Rica, and Santos and three roasted Robusta coffee beans from Togo, India, and Vietnam. The study identified 42 VOCs in each blend, including the six VOCs target of this work. Table S2 reports the data from Mondello et al. for the target compounds investigated in this study, where the contribution of each VOC in the six mixtures is represented by its mean relative percentage chromatographic peak area with respect to the total peak area of all detected VOCs in each coffee blend.

Table S2. Mean relative percentage peak areas for the six target molecules in each investigated mixture [21]. Values in bold are the maximum values for each molecule, which are used later for normalization.

Molecule	M1	M2	M3	M4	M5	M6
Pyridine	7.7	5.2	**7.9**	5.5	5.1	4.7
2-Methylpyrazine	6.8	6.1	5.9	6.1	8.2	**8.6**

2,5-Dimethylpyrazine	1.8	2.2	2.0	1.9	**3.7**	3.4
Furfural	**3.9**	3.5	1.9	1.4	1.5	1.9
5-Methylfurfural	**6.1**	6.0	3.9	2.0	2.6	2.9
Furfuryl Alcohol	17.8	19.4	**20.6**	12.0	10.4	8.9

These values were obtained with the HS-SPME–GC–MS technique: VOCs are extracted from the sample headspace using a coated fiber, which selectively absorbs the compounds before releasing them into the GC–MS system. Therefore, the quantification of each VOC in a mixture depends on the different adsorption interaction of the VOC with the fiber coating.

Since extraction efficiencies are not uniform for the different molecules, it is not possible to directly use the data in Table S2 as absolute concentration of the different VOCs in the mixture. Therefore, to realize reliable mixtures, the data in Table S2 are first normalized for each molecule, to compare the percentage content of a fixed VOC in the six mixtures. The normalized values, reported in Table S3, are then multiplied by the average expected headspace volume (Table S1) to retrieve realistic volumes for each component.

Table S3. Mean relative percentage peak areas normalized for each molecule, in each of the six examined mixtures.

Molecule	M1	M2	M3	M4	M5	M6
Pyridine	0.97	0.65	1	0.70	0.64	0.59
2-Methylpyrazine	0.79	0.70	0.68	0.71	0.95	1
2,5-Dimethylpyrazine	0.49	0.59	0.54	0.52	1	0.91
Furfural	1	0.89	0.5	0.36	0.37	0.50
5-Methylfurfural	1	0.98	0.65	0.33	0.43	0.47
Furfuryl Alcohol	0.86	0.94	1	0.58	0.51	0.43

The final volumes of each molecule, which have been experimentally injected to simulate the spectral signature of the six blends, are shown in Table S4.

Table S4. Final volumes for each molecule in each of the 6 investigated mixtures, expressed in nanoliters.

	**Volume (nl)**					
**Molecule**	**M1**	**M2**	**M3**	**M4**	**M5**	**M6**
Pyridine	944	635	971	680	619	572
2-Methylpyrazine	139	124	121	125	167	177
2,5-Dimethylpyrazine	47	57	52	50	96	87
Furfural	120	107	60	44	44	60
5-Methylfurfural	31	30	20	10	13	14
Furfuryl Alcohol	91	100	106	62	53	46
